# Difference in clinical presentation, immunology profile and treatment response of type 1 autoimmune hepatitis between United Kingdom and Singapore patients

**DOI:** 10.1007/s12072-016-9727-4

**Published:** 2016-04-21

**Authors:** Nwe Ni Than, Doreen Koay Siew Ching, James Hodson, Patrick McDowell, Jake Mann, Ravi Gupta, Ennaliza Salazar, Jing Hieng Ngu, Ye Htun Oo

**Affiliations:** Centre for Liver Research and NIHR Biomedical Research Unit in Liver Diseases, Institute of Immunology and Immunotherapy, University of Birmingham, Vincent Drive, Edgbaston, Birmingham, B15 2TT UK; Liver and Hepatobiliary Unit, Queen Elizabeth Hospital, Birmingham, UK; Department of Gastroenterology and Hepatology, Singapore General Hospital, Singapore, Singapore; Institute of Translational Medicine, Queen Elizabeth Hospital, Birmingham, UK; Leeds General Infirmary, Leeds, UK

**Keywords:** Autoimmune hepatitis, Asian, Caucasian, Ethnicity, Clinical features, Immunology, Survival

## Abstract

**Background:**

Autoimmune hepatitis (AIH) is an immune-mediated liver disease of unknown etiology. Increasing incidence of AIH in Asian patients has been reported. However, the phenotypic difference of Asian patients in Europe and Asia has still not been explored.

**Aim:**

To evaluate the clinical presentation, biochemical and immunological profiles, treatment response and survival outcome of type 1 AIH from two tertiary liver transplant centres (United Kingdom and Singapore).

**Method:**

Patients who fulfilled the simplified diagnostic scoring criteria of AIH were included in the study. Patients with overlap syndrome were excluded.

**Results:**

Totals of 40 Asian patients and 159 Caucasian patients from the University Hospital of Birmingham National Health Service Foundation Trust, UK, were compared with 57 Asian patients from Singapore General Hospital, Singapore. Asian patients from Singapore present significantly much later (median 55 vs. 32 years, *p* < 0.001), had higher MELD (*p* < 0.001) with lower albumin (*p* < 0.001) and higher bilirubin (*p* < 0.001) and lower ASMA positivity (*p* < 0.001) at diagnosis compared to UK Asian. Jaundice at presentation was much higher in Singapore Asian patients compared to UK Asian (53 vs. 30 %) but cirrhosis at diagnosis was more common in UK patients. Associated autoimmune conditions were less commonly seen in Singapore Asians. Comparing between UK cohorts, Asian patients present at younger age and have higher IgG level compared to Caucasian. Overall, 5-year transplant-free survival in all three cohorts was similar (*p* = 0.846).

**Conclusion:**

We demonstrate that AIH patients from Singapore present at older age with jaundice and have a low positivity of SMA. Despite these differences, transplant-free survival is similar in the two groups.

## Introduction

Autoimmune hepatitis (AIH) is an immune-mediated liver disease of an unknown etiology with female preponderance [1]. It is a rare disease with a prevalence of 10–17 per 100,000 populations in Europe and a mean incidence of 1–2 per 100,000 person-years [1, 2]. Type 1 AIH is characterised by the presence of anti-nuclear antibodies (ANA) and anti-smooth muscle antibodies (ASMA) in serum and it affects all ages, although the majority of cases are seen mainly in adults [1, 3]. AIH is commonly associated with other autoimmune conditions.

It has been reported that ethnicity has an impact on the prevalence, clinical presentations, and the natural history of type 1 AIH [4–6]. Ethnicity-related prevalence of AIH was found to be significantly lower in Asians who reside in New Zealand when compared to Caucasians, despite being exposed to the same environment [7]. A previous study described that AIH in patients of African ethnicity presented with more aggressive disease at clinical presentation, were less likely to respond to conventional immunosuppressive treatment and resulted in worse liver morbidity-related outcomes compared to Caucasians [8]. Asian-American AIH patients also demonstrated more aggressive disease with poorer survival, and Hispanic populations had a higher prevalence of biopsy-proven cirrhosis at presentation compared to Caucasians [9]. A study in the UK in 2002 showed that non-European Caucasians (African, Asian and Arabic) presented with more severe forms of liver disease in all aspects with international criteria for a diagnosis of AIH, and they seemed to require higher levels of immunosuppression from earlier points after diagnosis [10]. Another study by Verma et al. [8] mentioned that black ethnicity, especially men, have more aggressive disease, and that they are less likely to respond to standard immunosuppression with a worse outcome than non-black ethnicity. Taken together, these reports suggest that ethnicity has an impact on the natural history of AIH.

In this study, we aim to investigate the difference in natural history, mode of presentation, immunological profiles, associated autoimmune diseases, response to immunosuppression and survival outcomes of Asian type 1 AIH who reside in two different continents; namely the United Kingdom (UK) and Singapore. In addition, we compare a Caucasian patient cohort to an Asian cohort within the same hospital in the UK.

## Patients and methods

### Study cohort

The details of patients with type 1 AIH who attended liver outpatients at two tertiary liver transplant centres: University Hospital of Birmingham, UK, and Singapore General Hospital, Singapore, were retrospectively collected and analysed. Data was obtained between the years 1995 and 2015 in the UK and between 2001 and 2014 in Singapore.

The UK cohorts were drawn from 203 patients with type 1 AIH. Of these, 40 patients of Asian ethnicity were included in the study and compared with 57 patients from Singapore. An additional cohort of 159 Caucasian patients from the same UK hospital was also compared to the UK Asian cohort. The remaining four patients from the UK were of different ethnicities and were not considered in this analysis. The total duration of follow-up was 10 years (median of 4 years, range 1 month to 18 years) for the UK patient cohort and 14 years for the Singapore patient cohort (median of 4 years, range 1 month to 11 years).

All patients were evaluated and diagnosed with type 1 AIH by hepatologists within our medical centres based on clinical, biochemical, and immunological parameters along with liver histology. All liver biopsies were reviewed and reported by dedicated liver histopathologists. Patients with a simplified AIH score of greater than or equal to six were included in the study. Patients with overlap syndrome (primary biliary cirrhosis or primary sclerosing cholangitis with type 1 AIH) were excluded from the study. Other liver conditions, such as metabolic liver diseases, viral hepatitis, alcoholic or non-alcoholic fatty liver diseases, were also excluded.

Data were collected thoroughly from electronic case notes, clinical letters and treatment charts. Demographics data, clinical presentations, blood parameters such as biochemistry and immunology, liver histology and the presence of other associated autoimmune conditions were documented. Treatment of autoimmune hepatitis such as usage of the steroid, azathioprine, other second-line immunosuppression and liver-related complications such as decompensation and development of hepatocellular carcinoma were also documented.

### Statistical analysis

Comparisons were made between the UK Asian cohort and both the Singapore Asian and UK Caucasian cohorts. Since the UK Asian cohort was included in both these analyses, all *p* values were Bonferroni-adjusted for two comparisons, to help control the type 1 error rate.

Dichotomous variables were compared between the cohorts using Fisher’s exact tests, with ordinal and continuous variables assessed using Mann–Whitney tests, and reported as medians and interquartile ranges (IQRs). The number of flare-ups were converted into rates per patient-year, in order to account for the differences in follow-up durations between the patients, with comparisons between the cohorts performed using the OpenEpi calculator [11]. Transplant-free survival was assessed using Kaplan–Meier curves, with a log-rank test used to compare between the cohorts.

All analyses were performed using IBM SPSS Statistics 22 (IBM, Armonk, NY, USA), with the exception of the comparison of flare-up rates. Patients with missing data were excluded on a per-analysis basis and *p* < 0.05 was deemed to be indicative of statistical significance throughout.

## Results

### Comparison between UK Caucasian and UK Asian AIH patient cohorts

A total of 159 Caucasian patients were compared against 40 Asian patients from the same hospital in the UK. The details of the comparison are shown in Tables 1 and 2. The majority of the patients were female (79 % in Caucasian vs. 75 % in Asian, *p* = 1.000), with Caucasian patients presenting with the disease at significantly later ages than Asians (median age of 51 vs. 32 years, *p* < 0.001). BMI was similar in the two groups, with a median of 26.2 in Caucasian versus 26.0 in Asian (*p* = 0.556). No significant differences were detected between the groups in the rates of hypertension (*p* = 0.648), type 2 diabetes (*p* = 0.442) or hypercholesterolemia (*p* = 0.776).

**Table 1:** Demographic and clinical comparison between the three cohorts

Factor	UK Asian (*n* = 40)	Singapore Asian (*n* = 57)	*p* value*	UK Caucasian (*n* = 159)	*p* value**
Demographics					
Age at start of follow-up	32.0 (22.0–52.4)	55.0 (50.0–64.0)	**<0.001**	50.5 (28.9–60.3)	**0.002**
Gender (female)	30 (75 %)	49 (86 %)	0.386	125 (79 %)	1.000
Body mass index	26.0 (22.3–28.0)	24.7 (21.2–28.1)	0.580	26.2 (23.4–31.5)	0.556
Biopsied at diagnosis	29 (73 %)	56 (98 %)	**<0.001**	119 (75 %)	1.000
Cirrhosis at diagnosis	16 (40 %)	12 (23 %)	0.154	81 (51 %)	0.576
Hepatocellular carcinoma	0 (0 %)	1 (2 %)	1.000	3 (2 %)	1.000
Hypertension	8 (20 %)	24 (42 %)	0.058	45 (28 %)	0.648
Type two diabetes	9 (23 %)	10 (18 %)	1.000	22 (14 %)	0.442
Hypercholesterolemia	3 (8 %)	16 (28 %)	**0.036**	6 (4 %)	0.776
Clinical presentation					
Jaundice	12 (30 %)	30 (53 %)	0.074	33 (21 %)	0.424
Decompensated at diagnosis	4 (10 %)	2 (4 %)	0.452	15 (9 %)	1.000
Bloods at diagnosis					
Albumin	40 (31–43)	31 (27–35)	**<0.001**	39 (34–43)	1.000
Bilirubin	19 (11–38)	49 (18–172)	**<0.001**	20^a^ (11–38)	1.000
INR	1.1 (1.0–1.3)	1.2 (1.1–1.3)	0.606	1.1 (1.0–1.2)	0.636
Model for end stage liver disease (MELD)	7 (6–10)	13 (9–18)	**<0.001**	6 (6–10)	0.324
Other autoimmune conditions					
Any gastro-intestinal	5 (13 %)	0 (0 %)	**0.020**	12 (8 %)	0.688
Any connective	7 (18 %)	6 (11 %)	0.746	33 (21 %)	1.000
Any endocrine	3 (8 %)	1 (2 %)	0.606	19 (12 %)	1.000
Any renal	2 (5 %)	0 (0 %)	0.336	1 (1 %)	0.208
Any haematology	6 (15 %)	1 (2 %)	**0.036**	5 (3 %)	**0.020**

Continuous variables are reported as medians and interquartile ranges, with numbers and rates quoted otherwise

*p* values are post hoc comparisons between the UK Asian * Singapore Asian or ** UK Caucasian cohorts. Mann–Whitney tests and Fisher’s exact tests were used, as applicable, with Bonferroni adjustment for two comparisons applied to the resulting *p* values. *p* values significant at *p* < 0.05 are shown in bold

^a^Based on *n* = 32, due to missing data

**Table 2:** Comparison between UK Caucasian and UK Asian: Immunology and treatment

Factor	UK Asian (*n* = 40)	Singapore Asian (*n* = 57)	*p* value*	UK Caucasian (*n* = 159)	*p* value**
Immunology profile					
Anti-nuclear antibody	32 (80 %)	53 (93 %)	0.134	121 (76 %)	1.000
Anti-neutrophil cytoplasmic antibody	5 (13 %)	2 (4 %)	0.248	30 (19 %)	1.000
Anti-smooth muscle (type 1) antibody	29 (73 %)	16 (28 %)	**<0.001**	125 (79 %)	0.810
Soluble liver antigen	0 (0 %)	1 (2 %)	1.000	0 (0 %)	1.000
Ig G	21.9 (15.5–30.6)	25.0 (16.7–33.3)	0.832	17.8 (12.7–24.7)	**0.032**
Ig M	1.9 (1.2–2.5)	1.5 (1.1–3.1)	1.000	1.6 (1.1–2.5)	0.700
Ig A	4.0 (2.1–5.2)	4.1 (3.2–5.6)	0.342	2.9 (1.9–4.3)	0.079
Medications at diagnosis					
Steroids	37 (93 %)	55 (98 %)	0.610	135 (85 %)	0.604
Azathioprine	20 (50 %)	19 (33 %)	0.282	79 (50 %)	1.000
Mycophenolate mofetil	6 (15 %)	0 (0 %)	**0.008**	12 (8 %)	0.424
Current medications					
Steroids	30 (75 %)	36 (63 %)	0.542	109 (69 %)	1.000
Azathioprine	25 (63 %)	19 (33 %)	**0.014**	91 (57 %)	1.000
Mycophenolate mofetil	7 (18 %)	0 (0 %)	**0.003**	28 (18 %)	1.000
Number of flare-ups (per patient-year)^a^	0.33^a^ (0.26–0.41)	0.22^a^ (0.17–0.29)	0.062^a^	0.24^a^ (0.22–0.28)	0.060^a^
Concerns with compliance	6 (15 %)	0 (0 %)	**0.008**	15 (9 %)	0.770

Continuous variables are reported as medians and interquartile ranges, with numbers and rates quoted otherwise

*p* values are post hoc comparisons between the UK Asian and * Singapore Asian or ** UK Caucasian cohorts. Mann–Whitney tests and Fisher’s exact tests were used, as applicable, with Bonferroni adjustment for two comparisons applied to the resulting *p* values. *p* values significant at *p* < 0.05 are shown in bold

^a^Reported as rates per patient-year and 95 % confidence intervals, with *p* values from the mid-*P* exact test

The most commonly observed type of associated autoimmune conditions were connective tissue disorders, (vasculitis, systemic lupus erythromatus, limited scleroderma, Sjogren’s disease, vitiligo, psoriasis or rheumatoid disorder), which affected similar proportions of Asians and Caucasians (18 vs. 21 %, *p* = 1.000). The only type of associated autoimmune conditions that differed significantly by ethnicity was those that were haematology-related (e.g. autoimmune hemolytic thrombocytopenia or autoimmune hemolytic anemia), which were present in 15 % of Asians, compared to 3 % of Caucasian patients (*p* = 0.020).

Rates of liver biopsy at the time of diagnosis were similar in the Asian and Caucasian groups (73 vs. 75 %, *p* = 1.000), as were the rates of cirrhosis (40 vs. 51 %, *p* = 0.576). Hepatocellular carcinoma was uncommon in both cohorts, with no cases in Asian patients and only 3 (2 %) in Caucasians. Around 10 % of patients in both cohorts presented with features of liver decompensation such as ascites, hepatic encephalopathy or variceal bleed at the time of diagnosis. The proportions of patients presenting with jaundice were similar in both groups (21 in Caucasian vs. 30 % in Asian, *p* = 0.424). The models for end-stage liver disease (MELD) score were also similar in the two cohorts (median 6 in Caucasian vs. 7 in Asian, *p* = 0.324). At diagnosis, both Caucasian and Asian patients had comparable levels of albumin (median 39 vs. 40, *p* = 1.000), bilirubin (20 vs. 19, *p* = 1.000) and INR (1.1 vs. 1.1, *p* = 0.636).

The majority of the patients had anti-nuclear antibody positivity at diagnosis, followed by positive anti-smooth muscle antibody (ASMA) and anti-neutrophil cytoplasmic antibodies (ANCA), with all three having similar rates in both groups. Immunoglobulin-G (Ig G) was found to be significantly higher in Asian cohort (median 21.9 vs. 17.8, *p* = 0.032).

**Fig. 1:**
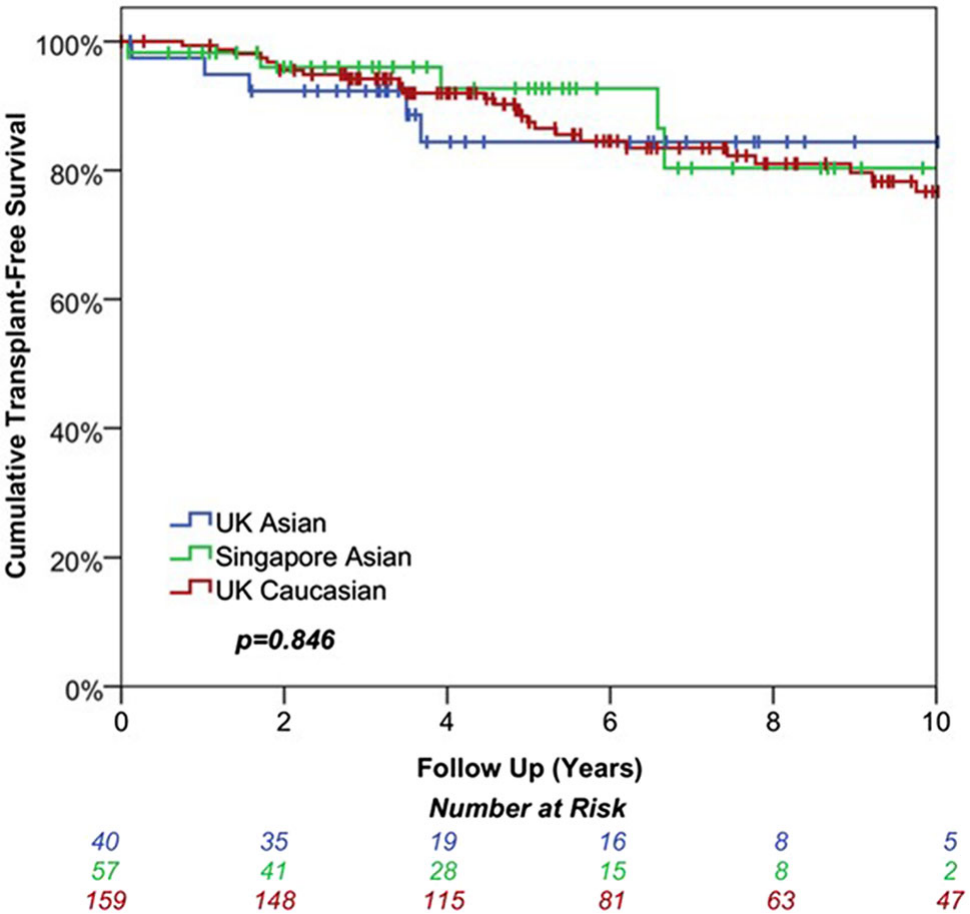
Kaplan–Meier survival curves of all study cohorts

The majority of the patients in both groups were started on steroids followed by azathioprine (AZA) and mycophenolate mofetil (MMF), with the treatment rates in both groups being similar (all, *p* = 1.000). There were only around a 20 % point reduction in use of steroids in both cohorts from the time of diagnosis to the most recent follow-up. The proportion of patients for whom there were concerns about compliance was 9 % in Caucasians and 15 % in Asians (*p* = 0.770). The documented number of flare-ups per year were low in both groups during follow-up (0.24 vs. 0.33 episodes per patient year). Transplant-free survival was also similar in the two groups, with rates at 5 years of 88 % in Caucasians compared to 84 % in Asians, (*p* = 1.000, Fig. 1).

### Comparison between UK Asian and Singapore Asian cohort of AIH patients

The 40 UK Asian patients were then compared to a cohort of 57 Asian patients from Singapore. The Singapore cohort presented at a significantly older age than the UK Asians (median 55 vs. 32 years, *p* < 0.001), and were significantly more likely to be biopsied at diagnosis (98 vs. 73 %, *p* < 0.001). No significant differences were detected in the gender (*p* = 0.386) or BMI (*p* = 0.580) distributions of the cohorts nor in the rates of type 2 diabetes (*p* = 1.000). However, Singapore Asians had significantly higher rates of hypercholesterolemia (28 vs. 8 %, *p* = 0.036) than UK Asians. Jaundice (53 vs. 30 %, *p* = 0.074) and hypertension (42 vs. 20 %, *p* = 0.058) were also more common in Singapore Asians, although not significantly so.

Of the blood work taken at diagnosis, the patients from Singapore had significantly lower albumin (median 31 vs. 40, *p* < 0.001), and significantly higher bilirubin (49 vs. 19, *p* < 0.001) and MELD (13 vs. 7, *p* < 0.001) than Asians from the UK. Associated autoimmune conditions were less commonly seen in Singapore Asians, with significantly lower rates of gastro-intestinal- (0 vs. 13 %, *p* = 0.020) and hematology-related (2 vs. 15 %, *p* = 0.036) conditions than Asians from the UK. Analysis of the immunological profiles of the two groups generally found no significant differences, with the exception of anti-smooth muscle antibody, which was detected in 28 % of Singapore Asians, compared to 73 % of those from the UK (*p* < 0.001).

Steroid usage was similar in the two groups at baseline (Singapore: 98 vs. UK: 93 %, *p* = 0.610) and the most recent follow-up (75 vs. 63 %, *p* = 0.542). Azathioprine was less commonly used in Singapore, with usage rates of 33 versus 50 % (*p* = 0.282) at diagnosis, and 33 versus 63 % (*p* = 0.014) at the most recent follow-up. None of the Singapore Asians used MMF, compared to 15 % of UK Asians at diagnosis and 18 % at the most recent follow-up (*p* = 0.008, 0.003). There were no concerns with compliance in the Singapore cohort, compared to 15 % of the UK Asian cohort (*p* = 0.008).

Transplant-free survival was similar in the two groups (*p* = 1.000), with rates at 5 years of 93 % in Singapore and 84 % in UK Asians (*p* = 1.000, Fig. 1). Flare-up rates were low in both groups, with an average of 0.22 per patient-year in Singapore Asians, compared to 0.33 in those from the UK (*p* = 0.062).

## Discussion

Ethnic difference has been reported to have an impact on the natural history of AIH [4–6, 8, 12]; however, the difference in clinical phenotype of Asian patients between different continents is still unexplored. In this study, we demonstrated differences in age at initial diagnosis, clinical presentation, immunology profiles, associated autoimmune conditions, immunosuppression used and treatment response/compliance, between Asian type 1 AIH patients in UK and Singapore.

Autoimmune hepatitis could present at any age but generally peaks around puberty and between the 4th and 6th decades in adult life [6, 13]. In our study, UK Caucasian patients presented at a significantly later age compared to the UK Asian population. From the comparisons with the Singapore Asian cohort, we observed that the UK Asian patients’ cohort presented earlier, on an average during their 3rd decade, compared to the 5th decade of the cohort from Singapore. This later presentation may explain the reason of higher incidence of hypertension and hypercholesterolemia in the Singapore Asian AIH cohort. AIH patients are generally asymptomatic, and diagnosis was made from routine blood tests, although jaundice at initial presentation is not uncommon. Interestingly, we noted that Singapore Asian patients commonly presented with jaundice, and had significant higher levels of bilirubin, a higher MELD score, and lower levels of albumin at diagnosis.

Immunological parameters such as hyperglobulinemia and positive autoantibodies are crucial for the diagnosis of type 1 AIH [14]. Type 1 AIH is characterised by the presence of ANA and/or SMA, although 19 % of AIH patients may not have any evidence of serological positivity at the time of presentation [15]. Low levels of SMA positivity have been previously described in Asian patients with type 1 AIH [16] and we have seen that in our Singapore Asian cohort. A large proportion of UK-Asian patients in our study were cirrhotic (40 %), thus there was a potential early onset of disease in the UK cohort, which may be attributable to a higher index of diagnosis related to higher SMA positivity on immunological investigation. UK Caucasian patients expressed lower levels of Ig-G compared to the Asian cohort, and were less likely to have associated hematological autoimmune conditions. None of the other factors compared between UK Asian and UK Caucasian were found to differ significantly, including transplant-free survival.

In addition, associated autoimmune diseases such as coeliac disease and inflammatory bowel disease are more common in UK Asian patients than those from Singapore. Previous observations have suggested that Th17 cells and memory mucosa lymphocytes are involved in gut–liver axis immunology [17, 18], and the high prevalence of inflammatory bowel disease in western countries may be the reason for the association.

Despite the availability of effective treatment, AIH is not a benign condition, with recent long-term studies reporting a twofold higher mortality than that of the general population [19, 20]. The majority of patients with AIH usually respond to standard immunosuppressive therapy with steroids and azathioprine [AZA] [21], which we observed in our cohort. Around 80 % of patients achieved remission with standard immunosuppressive therapy [22, 23], while 10–15 % of patients do not achieve biochemical remission with these standard therapies [24–26]. Alternative immunosuppression such as MMF could be used for those who cannot tolerate AZA [27]. Our study found that MMF was used as second-line therapy only in UK patients. All patients from Singapore tend to be controlled by standard immunosuppression with steroids and azathioprine.

End-stage liver cirrhosis related to AIH accounts for 4–6 % of adult liver transplantation in Europe and the United States [28, 29]. Of patients with AIH, 23 % from Singapore and 40 % of Asians and 51 % of Caucasians in the UK were cirrhotic at the time of presentation. Wong and colleagues reported that AIH in Asian patients who reside in United States tend to present with more aggressive states and patients are cirrhotic at the time of presentation [30]. In our study, the index incidence of cirrhosis at diagnosis was higher among UK Asian patients, which may be related to earlier onset or a high index of suspicion or early referral of this group to tertiary transplant centres.

Wong et al. [30] also suggested that INR tends to be higher in Asian population. However, we did not notice any difference in albumin titre and INR between the UK Asians and Caucasians in our study, as the majority of cases are compensated Child-Pugh grade A cirrhosis. Only 10 % of UK Asian patients and 4 % of Singapore patients presented with decompensated liver disease. It is possible that differences in health care provision between different studies may have impacted on the synthetic function and timing of presentation. Long-term liver-related morbidity such as decompensation or hepatocellular carcinoma incidence of both groups remained similar. Higher MELD score was observed in Singapore Asian patients, which could be related to higher bilirubin in this cohort, as the initial presentation in more than half of this cohort was with jaundice. Our study importantly demonstrated similar transplant-free survival in both groups.

We have identified a few limitations associated with our study, mainly due to the nature of retrospective and descriptive studies. First, we compared our UK Asians with Singapore Asians. The majority of UK Asian cohorts were of Indian origin while Singapore Asians were of Far East origin. Secondly, our cohort might represent AIH patients with more severe disease since we were tertiary liver transplant centres. Diet might also play an important role in differences between our cohorts. There is a population-based study conducted in New Zealand [31] that showed alcohol consumption was associated with a lower risk of diagnosing with AIH, but individuals who were vegetarian for more than a year and had antibiotics usage within 12 months before AIH diagnosis were significant risk factors associated with AIH.

In conclusion, our data suggested that AIH patients from Singapore present at older age with jaundice and have a low positivity of SMA with similar survival outcomes. These findings highlight that future work is required to explore the ethnicity-related epigenetic, microbiome, environmental and dietary factors for further understanding of AIH immunopathogenesis. We aim to conduct a large number multiple-centre study in the near future to have a better understanding of the impact of ethnicity in autoimmune hepatitis.
